# Correction of the tumor suppressor Salvador homolog-1 deficiency in tumors by lycorine as a new strategy in lung cancer therapy

**DOI:** 10.1038/s41419-020-2591-0

**Published:** 2020-05-21

**Authors:** Zhe Zhao, Shufen Xiang, Jindan Qi, Yijun Wei, Mengli Zhang, Jun Yao, Tong Zhang, Mei Meng, Xiaohua Wang, Quansheng Zhou

**Affiliations:** 1 Cyrus Tang Hematology Center, Jiangsu Institute of Hematology, Key Laboratory of Thrombosis and Hemostasis, Ministry of Health, 2011 Collaborative Innovation Center of Hematology, Soochow University, Suzhou, Jiangsu 215123 P. R. China; 2 School of Nursing, Soochow University, Suzhou, Jiangsu 215006 P. R. China; 3 Department of General Surgery, The First Affiliated Hospital of Soochow University, Suzhou, Jiangsu 215006 China; 4 State Key Laboratory of Radiation Medicine and Protection, School of Radiation Medicine and Protection, Soochow University, Suzhou, Jiangsu 215123 P. R. China; 5 Key Laboratory of Stem Cells and Biomedical Materials of Jiangsu Province and the Chinese Ministry of Science and Technology, Soochow University, Suzhou, Jiangsu 215123 P. R. China

**Keywords:** Cancer, Drug discovery

## Abstract

Salvador homolog-1 (SAV1) is a tumor suppressor required for activation of the tumor-suppressive Hippo pathway and inhibition of tumorigenesis. SAV1 is defective in several cancer types. SAV1 deficiency in cells promotes tumorigenesis and cancer metastasis, and is closely associated with poor prognosis for cancer patients. However, investigation of therapeutic strategies to target SAV1 deficiency in cancer is lacking. Here we found that the small molecule lycorine notably increased SAV1 levels in lung cancer cells by inhibiting SAV1 degradation via a ubiquitin–lysosome system, and inducing phosphorylation and activation of the SAV1-interacting protein mammalian Ste20-like 1 (MST1). MST1 activation then caused phosphorylation, ubiquitination, and degradation of the oncogenic Yes-associated protein (YAP), therefore inhibiting YAP-activated transcription of oncogenic genes and tumorigenic AKT and NF-κB signal pathways. Strikingly, treating tumor-bearing xenograft mice with lycorine increased SAV1 levels, and strongly inhibited tumor growth, vasculogenic mimicry, and metastasis. This work indicates that correcting SAV1 deficiency in lung cancer cells is a new strategy for cancer therapy. Our findings provide a new platform for developing novel cancer therapeutics.

## Introduction

The Hippo pathway is one of ten key signal pathways that control carcinogenesis^1–3^. In cancer contexts, the Hippo pathway bilaterally regulates tumorigenesis by activating either tumor suppressors or oncogenes in the pathway. Activation of the upstream tumor suppressors Salvador homologue 1 (SAV1, also known as WW45)^4^, mammalian Ste20-like 1 and 2 (MST1/2)^5^, large tumor suppressor 1 and 2 (LATS1/2), and MOB kinase activators^6^ in the Hippo pathway inhibits tumorigenesis. In contrast, escalation of the downstream oncoproteins, Yes-associated protein (YAP)^7^ and transcriptional co-activator PDZ-binding motif (TAZ)^8^, in the pathway promotes tumorigenesis^9–11^. Deficiency of the tumor suppressors SAV1, MST1/2, LATS1/2, and MOB kinase activators in the Hippo pathway activates oncogenic YAP/TAZ and causes carcinogenesis^9–11^.

SAV1 is at the front of the Hippo signal pathway (SAV1-MST1/2-LATS1/2-YAP/TAZ) and is required for the pathway activation^12–16^. In the Hippo pathway, SAV1 directly binds to protein kinases MST1/2 and induces the kinase cascade that promotes phosphorylation of MST1/2, LATS1/2, and YAP/TAZ. Then, the phosphorylated YAP and TAZ are degraded via the ubiquitin–lysosome system^16–23^. Degradation of the oncoproteins inhibits YAP/TAZ-mediated oncogene expression and tumorigenic signaling, resulting in tumor suppression^24,25^. In addition, SAV1 directly interacts with AKT, a key signaling protein, and inhibits AKT protein phosphorylation, thereby diminishing tumorigenic AKT-mediated carcinogenesis^26–29^. Notably, SAV1 suppresses Hedgehog signaling in lung cancer^30^ and also induces cancer cell apoptosis^31^. Thus, SAV1 plays a crucial role in tumor suppression via Hippo pathway-dependent and -independent mechanisms^12,16,32–35^.

The SAV1 gene is infrequently mutated in human cancers; however, its expression in cancer tissue is frequently downregulated either epigenetically or post transcriptionally^15,16,27^. Both gene knockout and downregulated expression of SAV1 promote carcinogenesis^17,36,37^. Notably, SAV1 deficiency is closely associated with poor prognosis for cancer patients^38^. In contrast, SAV1 overexpression by transfecting cancer cells with SAV1 cDNA–plasmid inhibits tumorigenesis and improves survival of tumor-bearing mice^30^. However, there are no reported therapeutics that correct SAV1 deficiency in cancer.

In this study, we explored exogenous agents that increase SAV1 levels in cancer cells and found that lycorine can correct SAV1 deficiency in lung cancer. Lycorine is a small molecule derived from *Lycoris radiate* and has strong anticancer effects and low toxicity^18,39^. Lycorine markedly increased SAV1 levels in cancer cells by inhibiting SAV1 degradation via the ubiquitin–lysosome system. Lycorine-induced increases of SAV1 levels activated MST1 and triggered degradation of oncogenic YAP. Higher SAV1 levels also inhibited YAP-activated transcription of various oncogenic genes and tumorigenic AKT and NF-κB signal pathways. Together, these effects resulted in strong inhibition of tumor growth, vasculogenic mimicry, and metastasis in tumor-bearing mice. Our findings suggest a new strategy for effective cancer therapy.

## Materials and methods

### Materials

Lycorine was purchased from Chengdu Must Biotech Ltd. with a purity >98%. Monoclonal antibody against β-actin and various chemicals were from Sigma-Aldrich (St. Louis, MO). TaqTM DNA Polymerase was from TaKaRa Biotechnology Co. Ltd (6119). Revert Aid TM First Strand cDNA Synthesis Kit was from Vazyme (R312-01). Antibodies against YAP (1A12), rabbit monoclonal antibody to phosphorylated AKT473 (587F11), total AKT (11E7), total NF-κB (D141E2), phosphorylated NF-κB (93H1), RBP-J (D10A4), MST1, and YAP (#56612) were obtained from Cell Signaling Technology. Antibodies to SKP2 and Notch1 were purchased from Santa Cruz Biotechnology (SC-74477, SC-376403). All secondary antibodies were obtained from Thermo Scientific (A27041, 31320).

### Cell culture and lycorine treatment

Human lung cancer cell lines SPC-A-1 and A549 were cultured in DMEM supplemented with 10% fetal bovine serum (the complete medium), at 37 °C in a humidified atmosphere of 5% CO_2_ and treated with lycorine at the concentrations of 0–10 μM, as previously described.

### Cell proliferation assay

SPC-A-1, A549, and HBE Cells were seeded in a 96-well plate and incubated with series concentrations of lycorine (0–60 μM). After 3 days, 10 μl of the CTG solution (5 mg/ml) was added to each well and incubated for 10 min at 37 °C, then the dual fluorescence wavelength absorbance at 490 nm was measured using SpectraMax M5 multi-detection reader, and the IC50 was assessed by SPSS 16.0.

### Cell-invasion assay

The ability of cell invasion was detected via transwell assay as we described before^40^. SPC-A-1 and A549 cells in 200 μl of serum-free DMEM were seeded to the upper chamber, and 500 μl of the complete medium was added to the lower chamber. Then, lycorine at the concentration of 0–10 μM was added to the upper and lower chambers. After incubation for 24 h, the membrane of the chamber was strained with Wright–Giemsa solution, and photographed under a microscope.

### Wound-healing assay

A549 cells were seeded in six-well plats for 24 h, wounded with a 200-μl plastic tip. Then the cells were incubated in 2% FBS DMEM with lycorine at the concentrations of 0–10 μM for 24 h, stained with Giemsa solution, and photographed under a microscope.

### Tube-formation assay

The tumor cell formation of capillary structure in vitro was tested, as we previously described^41^. In brief, viable SPC-A-1 cells were pre-treated with lycorine at the concentrations of 0–5 μM for 24 h and transferred to each well of a 48-well plate containing 0.15 ml Matrigel matrix. After incubation at 37 °C, 5% CO_2_ for 16 h, the tubes were stained with Wright–Giemsa solution and photographed by OLYMPUS FSX-100 microscope.

### Matrigel plug assay

The Matrigel plug assay was performed, as we described before^42^. In brief, 0.5 ml of ice-cold Matrigel was mixed with 50 μl (2 × 10^6^) of either SPC-A-1 or A549 cells with or without 10 μg of lycorine, and subcutaneously injected into the midventral abdominal region of 6–8-week-old nude mice (*n* = 7). After 14 days, the plugs in the mice were excised, sectioned, stained by hematoxylin–eosin (H&E) solution, and photographed by an OLYMPUS FSX-100 microscope.

### Tumor xenograft mouse model and cancer metastasis model

Tumor xenograft assay was conducted in accordance with protocols approved by the Institutional Animal Care and Use Committee (IACUC) of Soochow University. In brief, 1 × 10^7^ human lung cancer SPC-A-1 cells were subcutaneously injected in 6-week-old female nude mice, and the mice were randomly divided into two groups, then were intraperitoneally injected with either lycorine in PBS (10 mg/kg) or PBS as a control daily for 28 days. Tumor volume was measured and calculated according to the formula: tumor volume = 0.5 × length × width^2^.

The lung cancer cell metastasis model was conducted by the approved protocols of the Institutional Animal Care and Use Committee (IACUC) of Soochow University. A549 cells (3 × 10^6^) were injected into 6-week-old female nude mice via the lateral tail vein. After 24 h, either lycorine in PBS (10 mg/kg/day) or vehicle PBS was intraperitoneally injected once a day. The body weight of the mice was recorded every other day. After lycorine treatment for 34 days, the mice were sacrificed after anesthetized, and their lungs were collected and analyzed by H&E staining.

### RT-PCR and quantitative real-time PCR

The total RNA was extracted from A549 and SPC-A-1 cells incubated with lycorine. The RT-PCR and quantitative real-time PCR were conducted as we described before^43^.

### Western blotting

Proteins from SPC-A-1 and A549 cells were extracted using the MPER Mammalian Protein Extraction Kit, and the western blotting was performed as previously described^40^. After the proteins were resolved by sodium dodecyl sulfate polyacrylamide gel electrophoresis (SDS–PAGE) with Tris–glycine running buffer and transferred to nitrocellulose membranes. Membranes were blocked with 5% nonfat milk and incubated with primary antibodies at 4 °C overnight, followed by incubation with HRP-coupled secondary antibody for 1 h at room temperature and the enhanced chemiluminescence detection reagents, and exposed to X-ray film.

### Co-immunoprecipitation (Co-IP)

Co-IP was carried out as previously described^40^. About 400 μl of the A549 cell lysates were incubated with primary monoclonal antibody (1:500) or mouse normal IgG as a control at 4 °C for 4 h, then further incubation with 20 μl of prewashed protein A/G agarose beads at 4 °C overnight with rotation. The immune complexes were released from the beads in SDS-loading buffer. The proteins were detected by western blotting as mentioned above.

### Statistical analysis

All results represent the mean ± SD. Differences between the groups were assessed by one-way ANOVA using GraphPad Prism 5. Statistical comparisons were performed using the Student’s *t* test, and the significance of differences was indicated as *P < 0.05 and **P < 0.01, ***P < 0.001.

## Results

For this study, we used lung cancer cell lines as a model to explore traditional Chinese medicinal herbs that can raise SAV1 expression levels in cancer cells. We found that a small molecule called lycorine (MW: 287.31) significantly inhibited the growth of several lung cancer cell lines. Lycorine has an IC50 value of 4.75 μM in SPC-A-1 cells and 4.03 μM in A549 cells, respectively. Immortalized normal human bronchial epithelial (HBE) cells were less sensitive to lycorine, and we observed an IC50 value of 15.02 μM in the cells (Supplementary Fig. S1a–c). Lycorine arrested lung cancer cell cycle in the G2/M phase (Supplementary Fig. S1d, e). In addition, lycorine significantly induced lung cancer cell apoptosis (Supplementary Fig. S1f). We conducted a series of experiments to study the role and mechanism of lycorine as an anti-lung cancer agent.

### Lycorine effectively inhibits lung cancer tumor growth and metastasis in xenograft mice

We investigated the anti-lung cancer effects of lycorine in vivo using three mouse models: (1) human lung cancer cell xenograft, (2) cancer metastasis, and (3) Matrigel plug assay. In the xenograft mouse model, nude mice (*n* = 7 each group) were subcutaneously injected with human lung cancer SPC-A-1 cells, then administered with daily intraperitoneal injections of lycorine at 10 mg/kg body weight or PBS as a control for 28 days. The results showed that tumor volume notably diminished (Fig. 1a), and tumor weight reduced by more than three-fold in the lycorine treatment group compared with the control group (Fig. 1b, c). Mouse body weight was not significantly different between the two groups (Supplementary Fig. S2a),

**Fig. 1:**
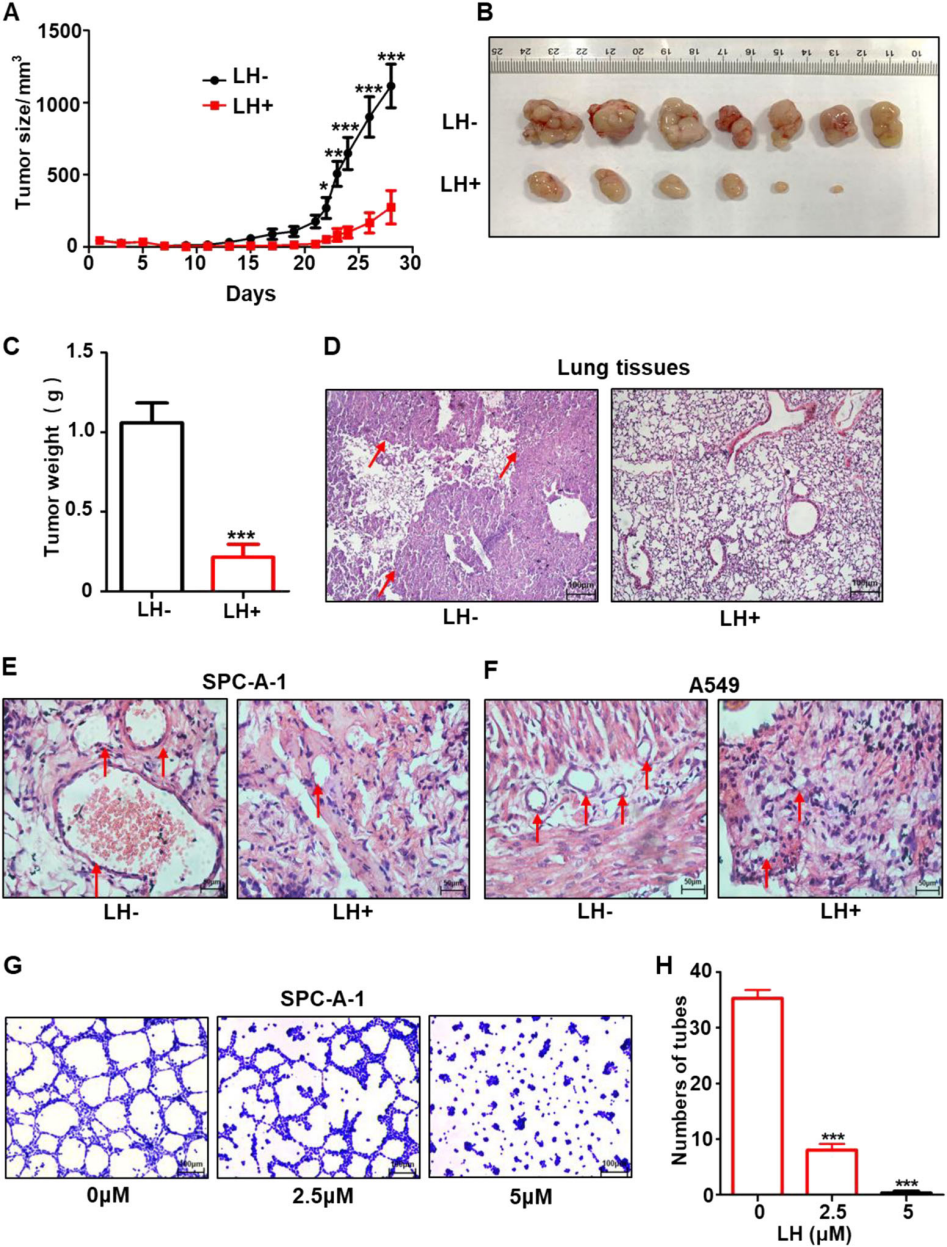
Lycorine effectively inhibits lung cancer tumor growth, vasculogenic mimicry, and metastasis in xenograft mice. The nude mice (*n* = 7 each group) were subcutaneously injected with 1 × 10^7^ human lung cancer SPC-A-1 cells, and then intraperitoneally injected with lycorine at the dose of 10 mg/kg/day or vehicle control daily. The tumor size (**a**) were recorded for 28 days. The excised tumors were imaged (**b**) and weighted (**c**). In the another cancer metastasis model, the nude mice (*n* = 7 each group) were daily treated with lycorine at the dose of 10 mg/kg/day or vehicle control daily, and the lung metastasis in either lycorine-treated or control mice were accessed by lung tissue H&E staining (**d**, ×200) arrowheads indicate lung cancer nodules in the lung tissues. LH + represents with lycorine treatment, LH− represents vehicle control. In the in vivo Matrigel plug assay model, lycorine effectively abrogated the formation of tumor blood vessels by SPC-A-1 (**e**) and A549 cells (**f**). The in vitro tumor cell tube-formation assay showed that lycorine at the concentration of 5 μM near completely inhibited SPC-A-1 cell formation of capillary like structure (tube) (**g**, **h**). Data represent the mean ± SD of triplicates. *P < 0.05. **P < 0.01. ***P < 0.001.

In the tumor metastasis mouse model, nude mice (*n* = 7 each group) received intravenous tail injections of A549 lung cancer cells. Mice were then intraperitoneally injected daily with lycorine at 10 mg/kg or PBS as a control for 34 consecutive days. After this time, we examined tumors in the lung tissues. H&E staining showed significantly less lung metastasis in the lycorine-treated group than the PBS control group (Fig. 1d). There were no significant differences between the lycorine-treated and PBS control groups for mouse body weight or organ coefficients (Supplementary Fig. S2b, c).

Lung cancer has robust vasculogenic mimicry to facilitate tumor growth and metastasis^44,45^. Therefore, we next investigated whether lycorine could inhibit tumor vasculogenic mimicry in mice. Using a Matrigel plug assay in nude mice (a classic method to assess both angiogenesis and vasculogenic mimicry in vivo) with A549 and SPC-A-1 lung cancer cells, we found that after 14 days of treatment, lycorine effectively abrogated the formation of tumor blood vessels in the mice (Fig. 1e, f). Lycorine did not affect mouse body weight (Supplementary Fig. S2d). Because tumor cell-mediated vasculogenic mimicry plays a crucial role in cancer dissemination^46–48^, we used an in vitro tube-formation assay to examine whether lycorine inhibited lung cancer cell-mediated tube formation. In this experiment, we observed that the tube-forming ability of lung cancer SPC-A-1 cells was markedly reduced by 5 μM lycorine treatment (Fig. 1g, h), suggesting that lycorine is a potent inhibitor of tumor vasculogenic mimicry. Collectively, these data indicate that lycorine markedly inhibited lung tumor growth, vasculogenic mimicry, and metastasis with low toxicity.

### Lycorine activates the tumor-suppressive Hippo signal pathway by increasing SAV1 levels in lung cancer cells

Our preliminary experiments of gene expression profile using reverse transcription polymerase chain reaction (RT-PCR) and western blotting showed that lycorine promoted SAV1 expression in the tumors of tumor-bearing mice. Because no effects of lycorine on the Hippo pathway in cancer is reported and the SAV1-targeted therapeutics against cancer is lacking, we studied the mechanisms of lycorine’s anti-lung cancer effect, focusing on the Hippo pathway. We observed that lycorine treatment markedly increased levels of SAV1 and activated MST1 in the Hippo pathway, but significantly decreased the level of oncogenic YAP in lung cancer tissues (Figs. 2–4). Immunohistochemical (IHC) staining showed that SAV1 expression levels were significantly higher in the tumor samples from the lycorine-treated xenograft mice compared with those of the PBS controls (Fig. 2a, b). Tissue immunofluorescent (IF) staining confirmed the higher SAV1 protein levels after lycorine treatment (Fig. 2c), suggesting that lycorine increased SAV1 levels in the tumors from the xenograft mice. In addition, IHC staining showed that levels of the oncogenic YAP protein were significantly lower in tumor tissues from lycorine-treated mice than in controls (Fig. 2d, e). To further test these findings, we used two different methods to measure SAV1 and YAP expression in the tumor samples. Western blotting indicated that SAV1 protein levels were 5.7 times higher in tissues from the lycorine-treated mice, and YAP protein levels were 4.2 times lower in the tumor tissues from the lycorine-treated mice compared with controls (Fig. 2f, g, h); whereas RT-PCR (Fig. 2i) and quantitative PCR (qPCR) (Supplementary Fig. S2e) found no difference in mRNA levels between these two groups. These data indicate that lycorine regulates expression of SAV1 and YAP post transcriptionally.

**Fig. 2:**
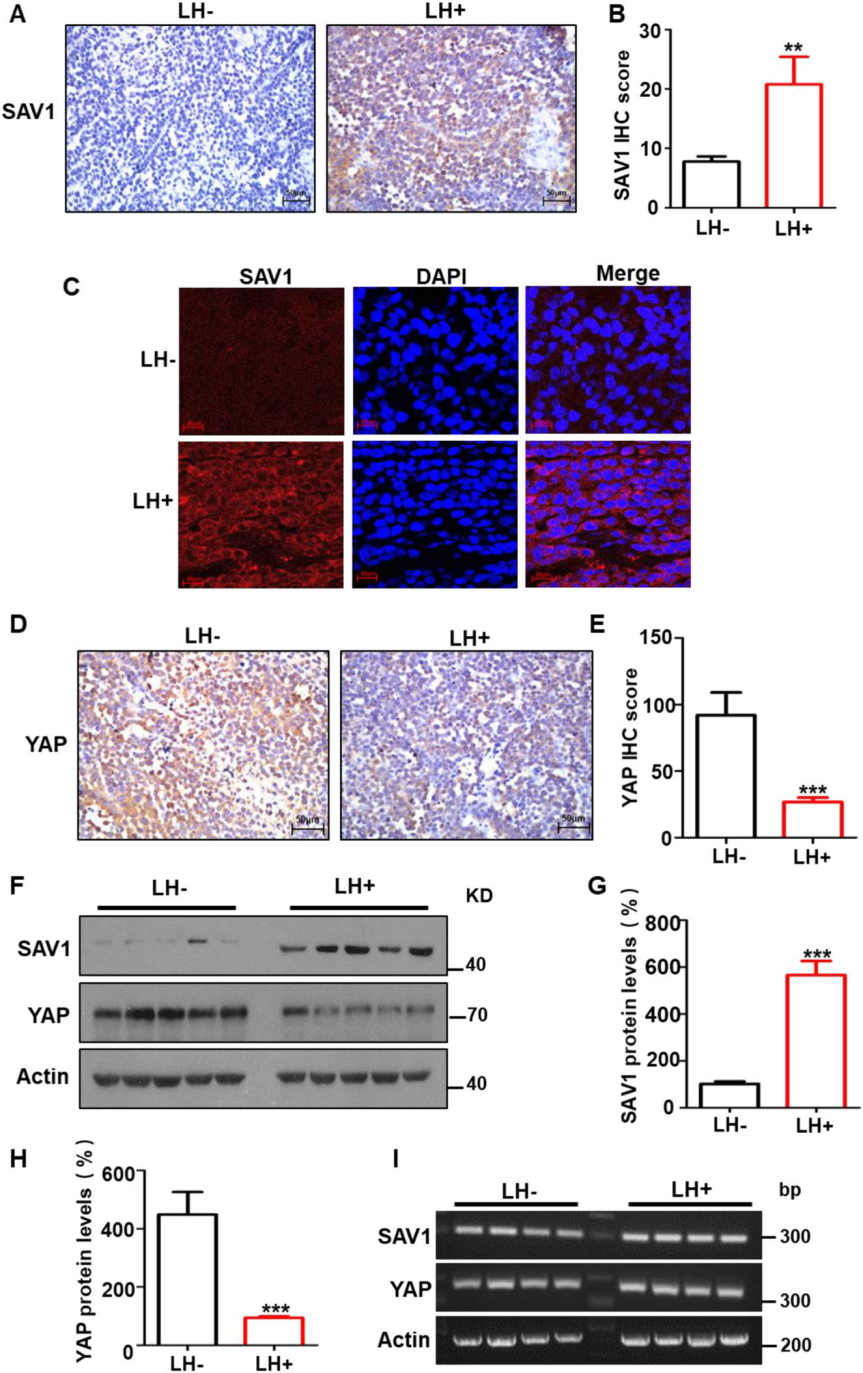
Lycorine elevates the tumor suppressor SAV1 levels and reduces the oncogenic YAP amounts in the tumors from xenograft mice. The levels of SAV1 and YAP proteins in the tumors from xenograft mice were detected by IHC and IF, respectively. Lycorine raised SAV1 levels (IHC: **a**, **b**, ×200; IF: **c**, ×1000), while YAP amounts were decreased by lycorine treatment (IHC: **d**, **e**). The amounts of SAV1, YAP, and β-actin in the tumor tissues from xenograft mice were detected by western blotting (**f**–**h**) and RT-PCR (**i**), respectively. LH + represents with lycorine treatment, LH− represents vehicle control. Data represent the mean ± SD of triplicates. *P < 0.05. **P < 0.01. ***P < 0.001.

**Fig. 3:**
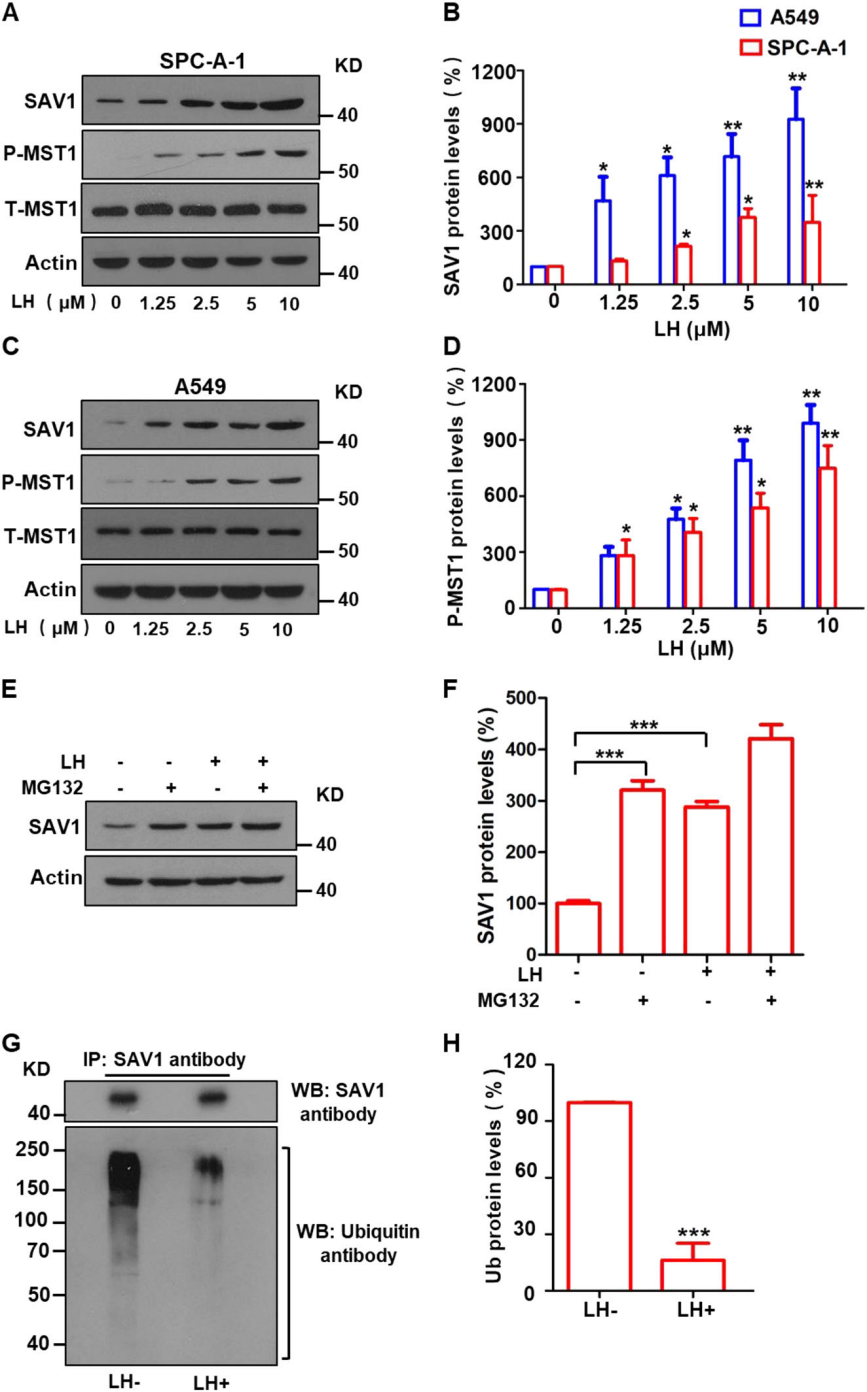
Lycorine significantly elevates SAV1 protein levels through degradation of the protein via ubiquitination-lysosome system. After A549 and SPC-A-1 cells were treated with lycorine (LH) at the concentrations of 0–10 μM for 72 h, the protein levels of SAV1, total MST1 (T-MST1), and phosphorylated MST1 (p-MST1) were determined by Western blotting. SAV1, p-MST1 protein levels were increased in lung cancer SPC-A-1 (**a**, **b**) and A549 (**c**, **d**) cells by lycorine in a dosage-dependent manner. Treatment of the lung cancer cells with the ubiquitin inhibitor MG132 significantly increased SAV1 protein levels, combination of lycorine with MG132 further increased SAV1 protein levels (**e**, **f**). Co-IP and western blotting analysis displayed that lycorine significantly decreased SAV1 protein ubiquitination (**g**, **h**). Data are shown as mean ± SD of three independent replicates.

**Fig. 4:**
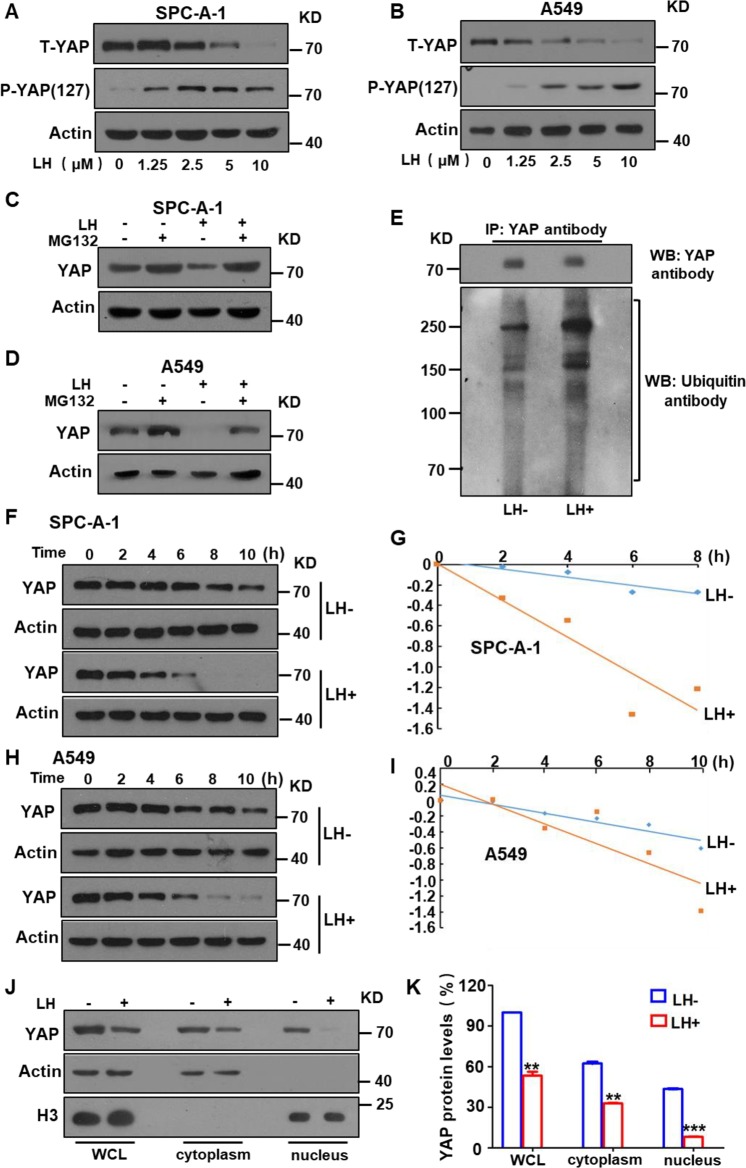
Lycorine promoted the phosphorylation and ubiquitin-mediated degradation of oncogenic YAP in lung cancer cells. Western blotting showed the at lycorine notably decreased total YAP (T-YAP) protein levels, but markedly increased the phosphorylation of the protein (p-YAP) in a dose-dependent manner in lung cancer SPC-A1 (**a**) and A549 cells (**b**). Ubiquitination inhibitor MG132 significantly reversed the lycorine-mediated YAP protein degradation in SPC-A-1 (**c**) and A549 cells (**d**). Lycorine significantly increased YAP protein ubiquitination in SPC-A-1 cells (**e**). The half-life of YAP was been shorten with lycorine treatment in the presence of the protein synthesis inhibitor CHX in SPC-A-1 (**f**, **g**) and A549 cells (**h**, **i**). The intracellular protein distribution analysis showed that lycorine decreased total and plasma YAP levels, and markedly reduced nuclear YAP levels (**J**, **k**). Data are shown as mean ± SD of three independent replicates.

Next, we studied the mechanisms of lycorine-induced increases of tumor-suppressive SAV1 and decreases of oncogenic YAP in the Hippo pathway using a lung cancer cell model. Western blotting showed that lycorine treatment not only markedly increased SAV1 protein levels (Fig. 3a–d; Supplementary Fig. S3a, b) but also dramatically increased phosphorylation of its interacting protein MST1, indicating MST1 protein activation, in SPC-A-1 (Fig. 3a, b) and A549 lung cancer cells (Fig. 3c, d) in a concentration-dependent manner. Lycorine treatment did not change total MST1 protein levels or SAV1 mRNA levels in these lung cancer cells (Supplementary Fig. S3c, d). These data suggest that the lycorine-induced increase in SAV1 protein activates tumor-suppressive MST1 in lung cancer cells.

The mechanisms of the effect of lycorine on SAV1 protein levels in lung cancer cells is unclear. We hypothesized that lycorine may prevent SAV1 protein from degradation. We found that treating SPC-A-1 cells with MG132, a ubiquitination–proteasome inhibitor, increased SAV1 levels and that lycorine treatment further increased SAV1 protein levels in cancer cells in the presence of MG132 (Fig. 3e, f). Furthermore, co-immunoprecipitation (Co-IP) and western blotting showed that levels of ubiquitinated SAV1 protein were markedly lower in lycorine-treated SPC-A-1 cells than in cells without lycorine treatment (Fig. 3g, h), suggesting that lycorine-induced SAV1 protein accumulation in lung cancer cells is mediated by inhibiting the ubiquitination–proteasome system that degrades SAV1.

### Lycorine promotes the degradation of oncogenic YAP and decreases expression of tumorigenic genes and cancer cell invasion

Oncogenic YAP, a key downstream protein in the Hippo pathway, promotes transcription of many tumorigenic genes and activates multiple signaling pathways^49–51^. We tested whether lycorine treatment affected YAP expression. Western blotting results showed that total YAP protein levels were notably reduced, but phosphorylated YAP protein (*p*-YAP) levels increased in a lycorine concentration-dependent manner in both SPC-A-1 (Fig. 4a) and A549 lung cancer cells (Fig. 4b). In addition, western blotting and Co-IP results showed that lycorine treatment increased YAP protein ubiquitination and degradation in the cancer cells via the ubiquitin–proteasome system (Fig. 4c, d, e). Furthermore, dynamic analysis of YAP degradation indicated that lycorine shortened the half-life of YAP in both SPC-A-1 (Fig. 4f, g) and A549 cells (Fig. 4h, i). Intracellular YAP protein localization analysis using nuclear protein extracts and western blotting showed that lycorine profoundly reduced the levels of nuclear YAP protein (Fig. 4j, k). These data indicate that lycorine reduced YAP protein levels by triggering phosphorylation and degradation of YAP, thereby reducing levels of oncogenic YAP in the cell nucleus. Nuclear YAP promotes the transcription of various oncogenic genes^10,52–54^. Therefore, we investigated the effect of lycorine treatment on the expression of YAP-activated transcription of oncogenic genes. qPCR showed that lycorine treatment significantly decreased the mRNA levels of two YAP-regulated tumor angiogenic genes, Sema4D and Ang2, in SPC-A-1 (Fig. 5a, c) and A549 cells (Fig. 5b, d). Lycorine treatment also decreased expression of S-phase kinase-associated protein 2 (SKP2), a YAP-controlled E3 ligase that is important for regulating ubiquitination and cell cycle (Fig. 5e, f). In addition, protein levels of Sema4D and SKP2 were significantly lower after lycorine treatment (Supplementary Fig. 4a–f). Because there is cross talk between the Hippo signaling pathway and the AKT and NF-κB signal pathways^43,55^, we investigated the effect of lycorine on these two signaling pathways. Our results showed that lycorine treatment significantly inhibited the phosphorylation of AKT and NF-κB in both SPC-A-1 (Fig. 5g, h) and A549 cells (Fig. 5i, j). Collectively, these data suggest that lycorine activates the tumor-suppressive Hippo pathway and inhibits the transcription of oncogenic genes and tumorigenic AKT and NF-κB signaling pathways.

**Fig. 5:**
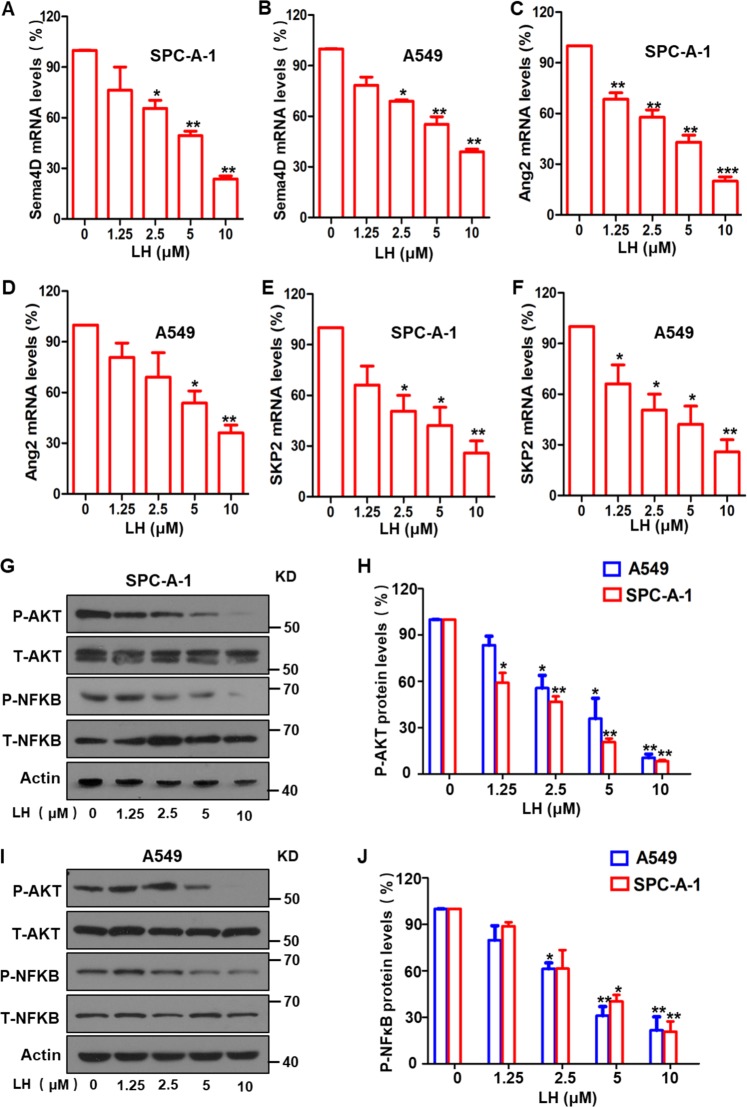
Lycorine inhibited YAP-promoted transcription of downstream oncogenic genes and the phosphorylation of AKT and NF-κB. Quantitative real-time PCR showed that treatment of the lung cancer SPC-A-1 and A549 cells with lycorine significantly reduced the levels of Sema4D (**a**, **b**), Ang2 (**c**, **d**), and SKP2 (**e**, **f**). Western botting showed that the phosphorylation of AKT (**g**, **h**) and NF-κB (**i**, **j**) was significantly inhibited by lycorine. Data are shown as mean ± SD of three independent replicates.

Malignant tumor cells are highly motile and easily metastasize to multiple organs^56,57^. Given our finding that lycorine inhibited lung cancer cell angiogenesis and dissemination (Fig. 1d–h), we next studied the effect of lycorine on lung cancer cell motility. A wound-healing assay showed that lycorine significantly decreased A549 cell migration (Fig. 6a, b) and markedly inhibited the invasion of both SPC-A-1 and A549 cells in a concentration-dependent manner (Fig. 6c–e). Furthermore, the invasion of SPC-A-1 cells was significantly reduced by YAP shRNA (Fig. 6f–i), and combining YAP shRNA with lycorine treatment further reduced YAP expression (Fig. 6f–i) and decreased the invasion capability of the cancer cells (Fig. 6j, k), suggesting that lycorine treatment was synergistic with a YAP inhibitor.

**Fig. 6:**
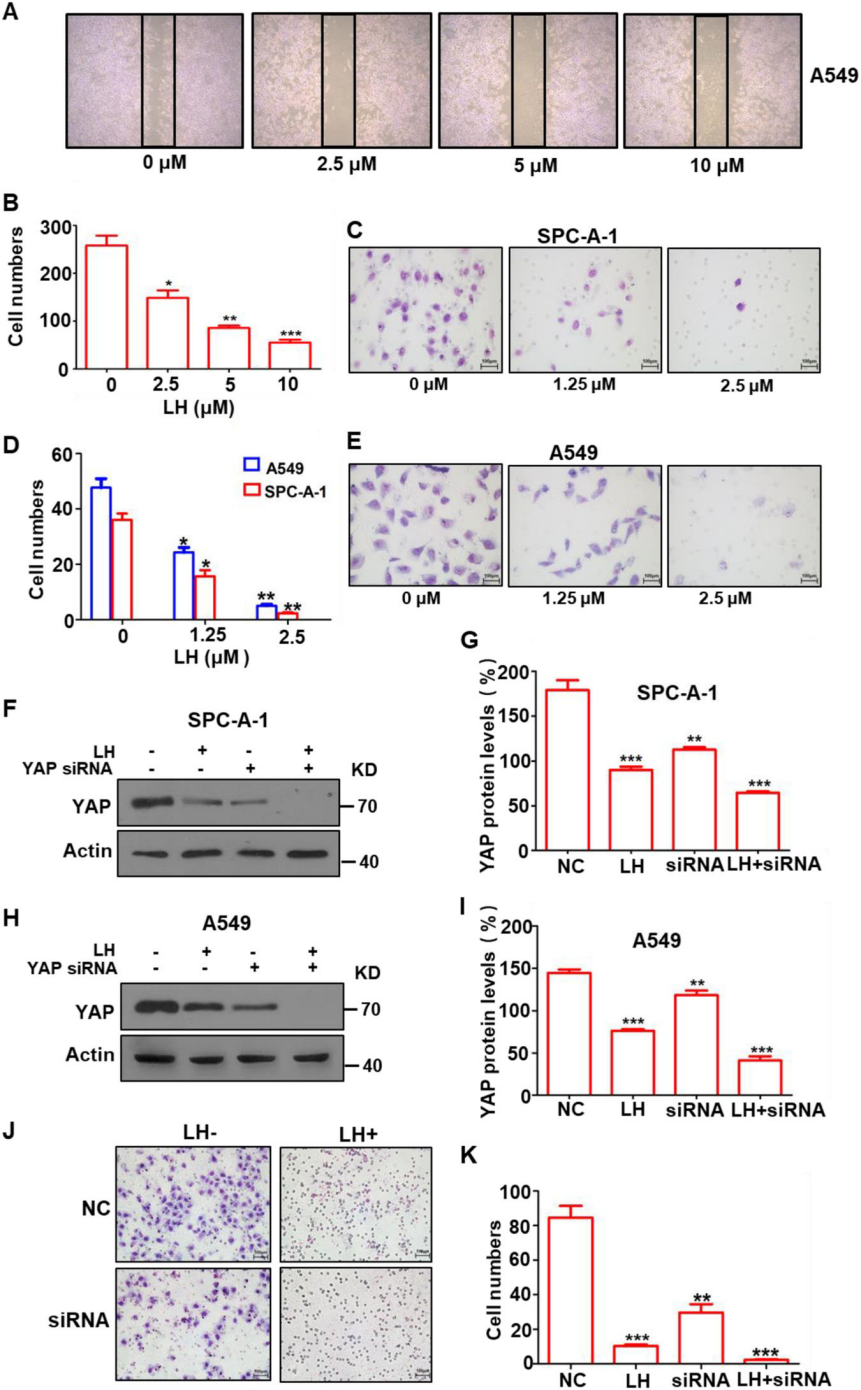
lycorine markedly suppressed YAP-mediated lung cancer cell migration and invasion. Lycorine significantly inhibited lung cancer A549 cell migration in a concentration-dependent manner in the wound-healing assay (**a**, **b**). Lycorine at a concentration of 2.5 μM markedly reduced the migration of SPC-A-1 (**c**, **e**) and A549 (**d**, **e**) cells invasion in a transwell analysis. Western blotting showed that YAP siRNA decreased YAP expression and combination of YAP siRNA with lycorine further reduced YAP expression in SPC-A-1 (**f**, **g**) and A549 cells ((**h**, **i**); meanwhile, YAP siRNA markedly diminished SPC-A-1 cell migration and combination of YAP siRNA with lycorine near completely suppressed further lung cancer cell migration (**j**, **k**). Data are shown as mean ± SD of three independent replicates.

Together, these data indicate that lycorine treatment suppresses tumorigenicity, angiogenesis, invasion, and metastasis of lung cancer cells primarily by increasing SAV1 levels. We hypothesize that SAV1 increase is due to decreased SAV1 protein ubiquitination and degradation, which activates downstream MST1. MST1 activation promotes the ubiquitination and degradation of oncogenic YAP and inhibits transcription of the YAP-target genes. As a result, AKT and NF-κB signal pathways are suppressed, which effectively inhibits proliferation, motility, vasculogenic mimicry, and metastasis of lung cancer cells, resulting in strong anticancer effects (Fig. 7).

**Fig. 7:**
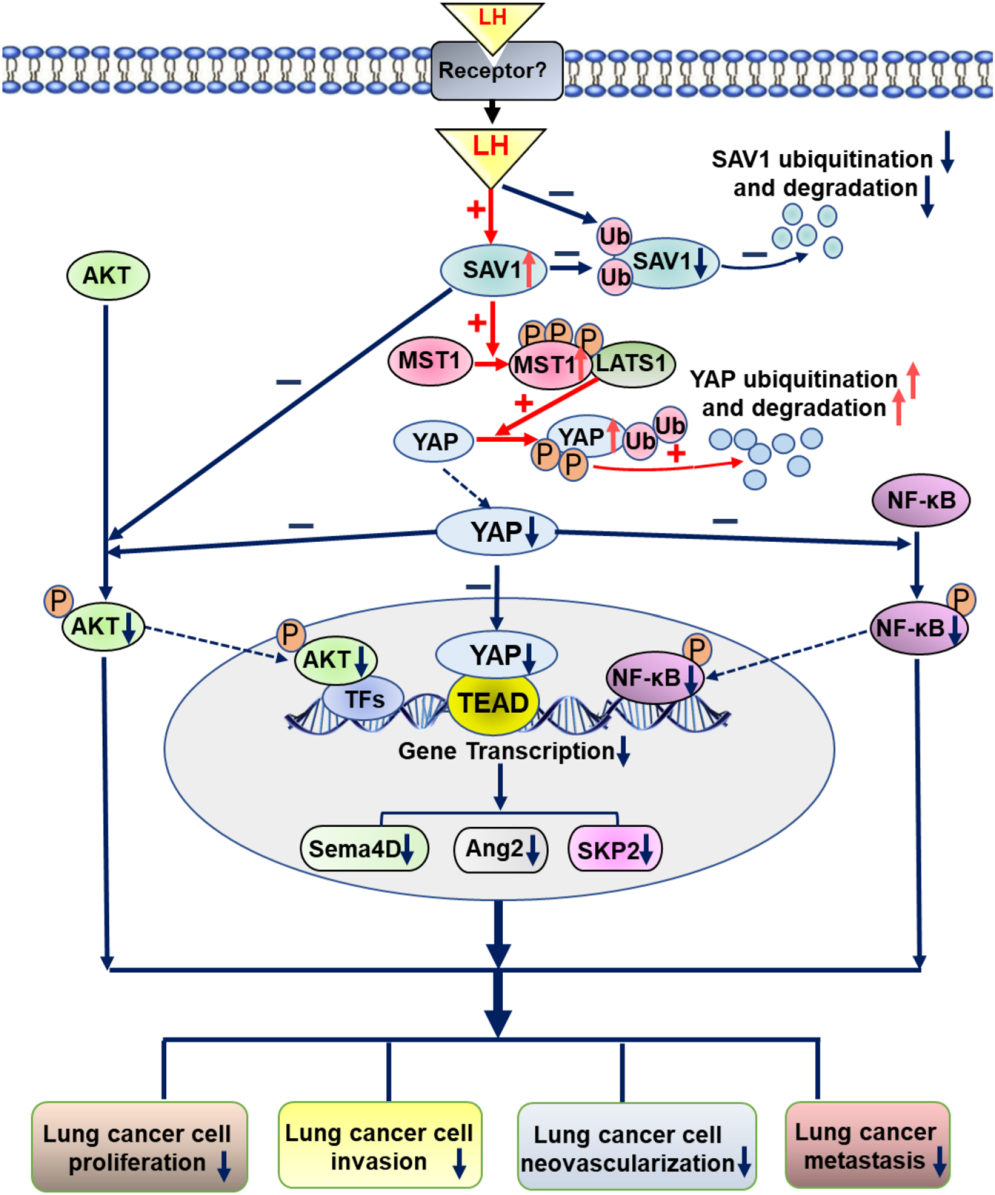
Schematic representation of correction of the SAV1 deficiency in lung cancer cells by lycorine as a new strategy for effective cancer therapy. Lycorine increases SAV1 levels through inhibiting SAV1 protein degradation via ubiquitination–proteasome system, induced MST1 phosphorylation, consequently reduces oncogenic YAP amounts by promoting the degradation of YAP protein and inhibits the transcription of YAP downstream oncogenic genes in lung cancer. In addition, elevation of SAV1 levels by lycorine inhibits AKT signaling and reduction of YAP suppresses NF-κB signaling, resulting in effective inhibition of lung cancer cell proliferation, motility, vasculogenic mimicry, and metastasis.

## Discussion

The tumor suppressor SAV1 expression is downregulated in various malignant tumors, including lung cancer^58,59^, and results in robust tumorigenicity^12,18^. In the Hippo pathway, SAV1 activates MST1/2-LAST1/2, a downstream complex that stimulates phosphorylation, ubiquitination, and degradation of oncogenic YAP^34,60^. The deficiency of SAV1 in cancer cells causes the MST1/2 proteins to be inactive. Therefore, upregulation of SAV1 expression and subsequent activation of the MST1/2-LAST1/2 complex in cancer cells are important treatment strategies to be explored. In this study, we observed that lycorine treatment increased SAV1 levels, which activated MST1 and promoted YAP degradation. We hypothesize that YAP degradation then downregulated the YAP downstream tumorigenic genes and suppressed AKT and NF-κB signaling pathways in lung cancer cells. Increasing SAV1 levels to activate the tumor-suppressive Hippo pathway and inhibition of these oncogenic pathways resulted in robust inhibition of tumorigenicity, invasion, and metastasis of lung cancer cells, suggesting that use of exogenous agents to increase SAV1 levels in cancer presents a new strategy for discovering novel anticancer drugs.

SAV1 has long been regarded as an adaptor protein in the Hippo pathway, but has not been well-studied, although emerging evidence indicates that SAV1 is essential for tumor suppression^16^. Our study found that SAV1 is an excellent target for cancer therapy, and this approach has several advantages. First, the tumor suppressor SAV1 is downregulated in a variety of malignant tumors^58,59^; thus, the strategy of increasing SAV1 expression in malignant tumors may be applied broadly to prevent and treat various types of cancer. Second, SAV1 induces the protein kinase-phosphorylation cascade (MST1/2-LAST1/2-YAP/TAZ) in the Hippo pathway and simultaneously inhibits several other tumorigenic signal pathways, such as the AKT and NF-κB signal pathways in cancer cells, therefore amplifying SAV1-mediated tumor suppression. Third, SAV1 overexpression inhibits cancer cell proliferation and motility and induces tumor cell apoptosis^13,61^ without obvious side effects in tumor-bearing mice. Thus, SAV1 is an excellent target for cancer therapy.

Lycorine treatment is observed to have the effects of antiviral, antimalarial, antibacterial, anti-inflammatory^62–65^, and anti-angiogenesis^39,66^ in multiple cancers^67–69^. Lycorine’s anticancer effect is mediated by suppressing expression of numerous oncogenic genes, such β-catenin^40^, m-TOR^70^, Erk^71^, and also by inhibiting multiple signaling pathways, including AKT^72^, Stat3^73^, Wnt-β-catenin^74^, Src-Fak^75^, and NF-κB. Lycorine also blocks EMT and has an anti-colon carcinoma effect^61,76^. In this study, we found that lycorine treatment powerfully activated the tumor-suppressive Hippo pathway, without obvious toxicity in the tumor-bearing mice at the doses tested. Thus, the strong anticancer effect and low toxicity of lycorine warrants further investigation into its development as a novel cancer therapy.

## Supplementary information

Supplementary figure 1

Supplementary figure 2

Supplementary figure 3

Supplementary figure 4

Supplementary figure legends
