# Supplementary figures and images for: Correction of the tumor suppressor Salvador homolog-1 deficiency in tumors by lycorine as a new strategy in lung cancer therapy
Source: Cell Death Dis. 2020 May 21;11(5):387. doi: 10.1038/s41419-020-2591-0 (PMC7242319; doi:10.1038/s41419-020-2591-0)

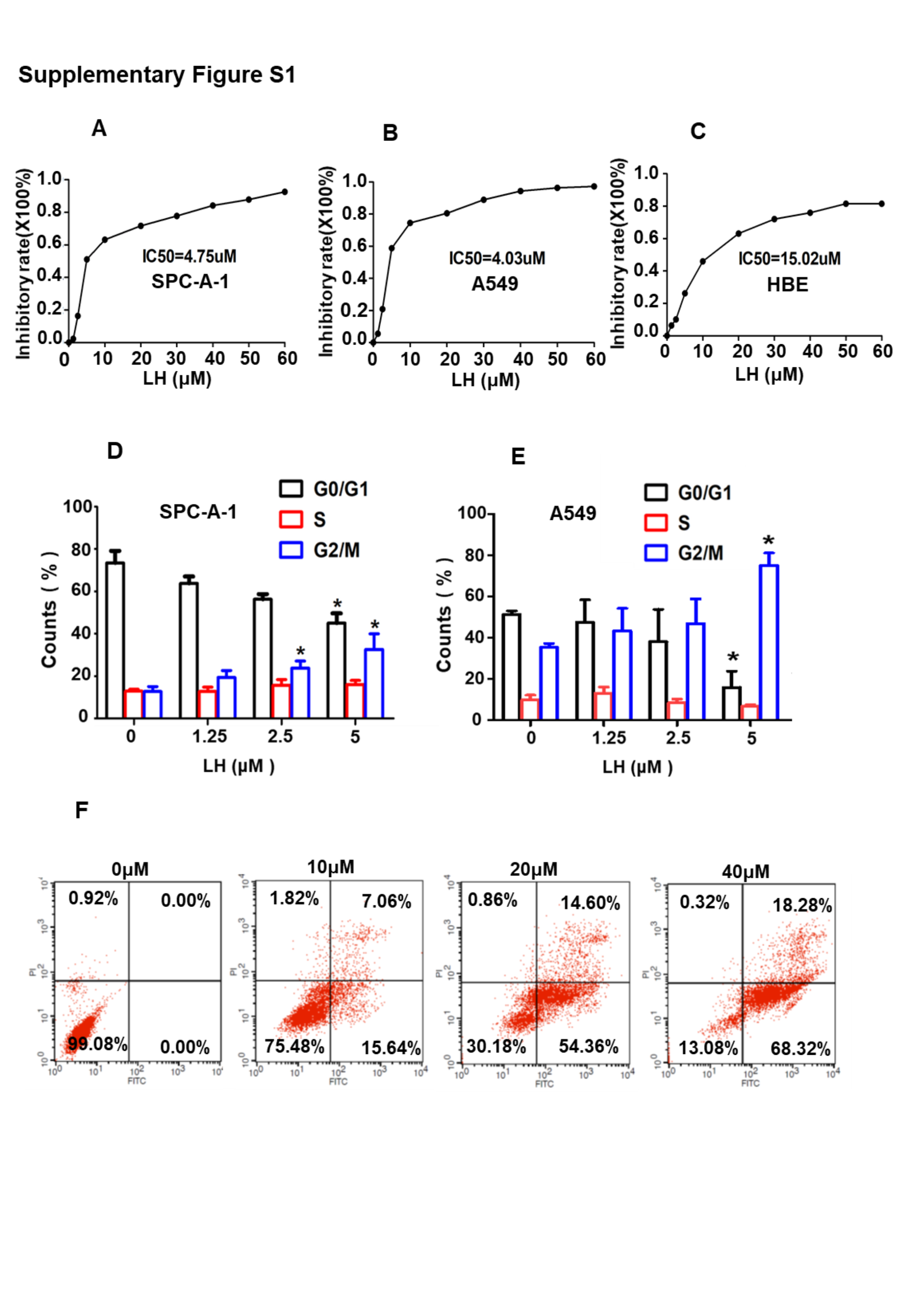

Supplement: Supplementary file 1 — Supplementary figure 1 [file 41419_2020_2591_MOESM1_ESM.tif]

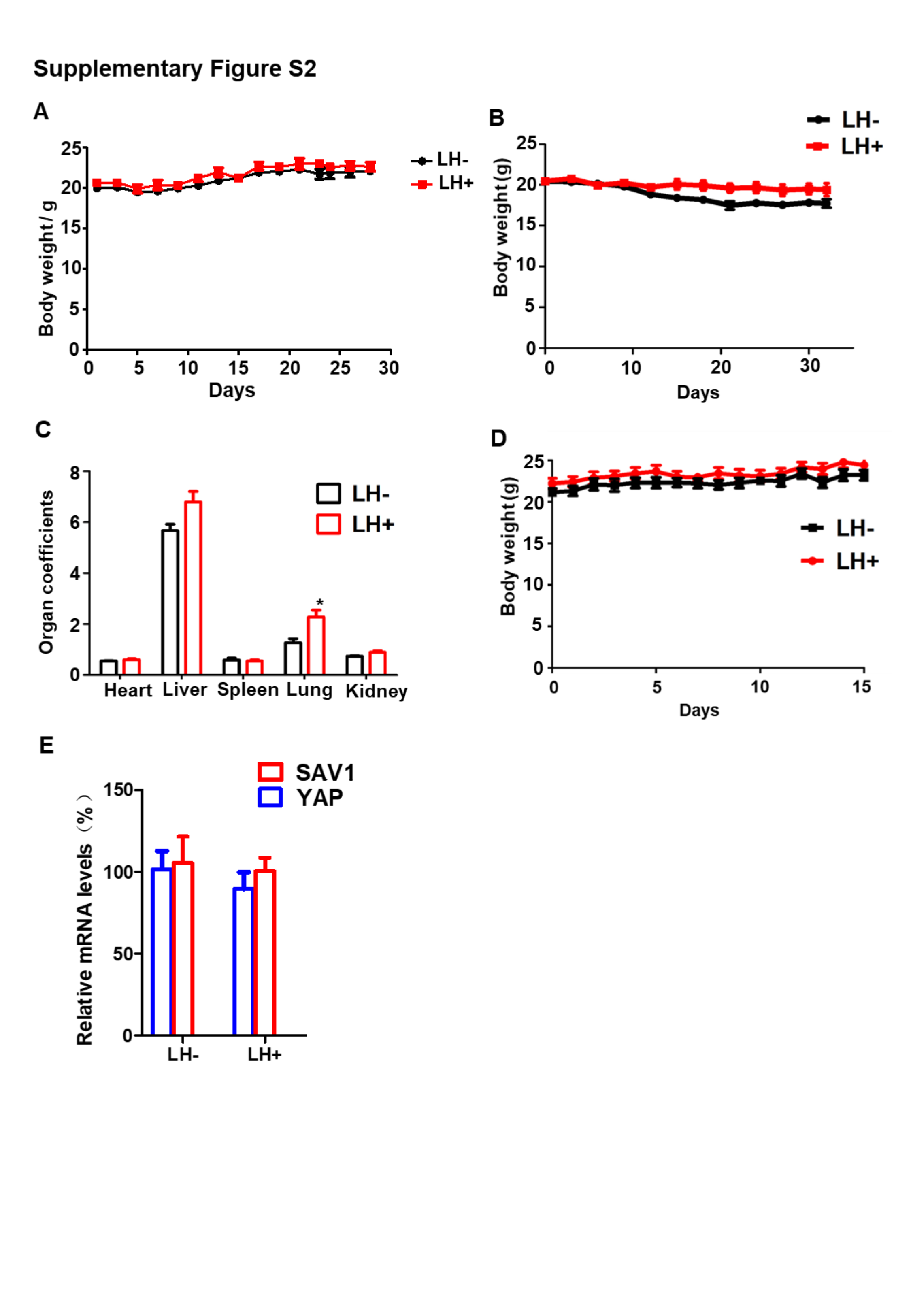

Supplement: Supplementary file 2 — Supplementary figure 2 [file 41419_2020_2591_MOESM2_ESM.tif]

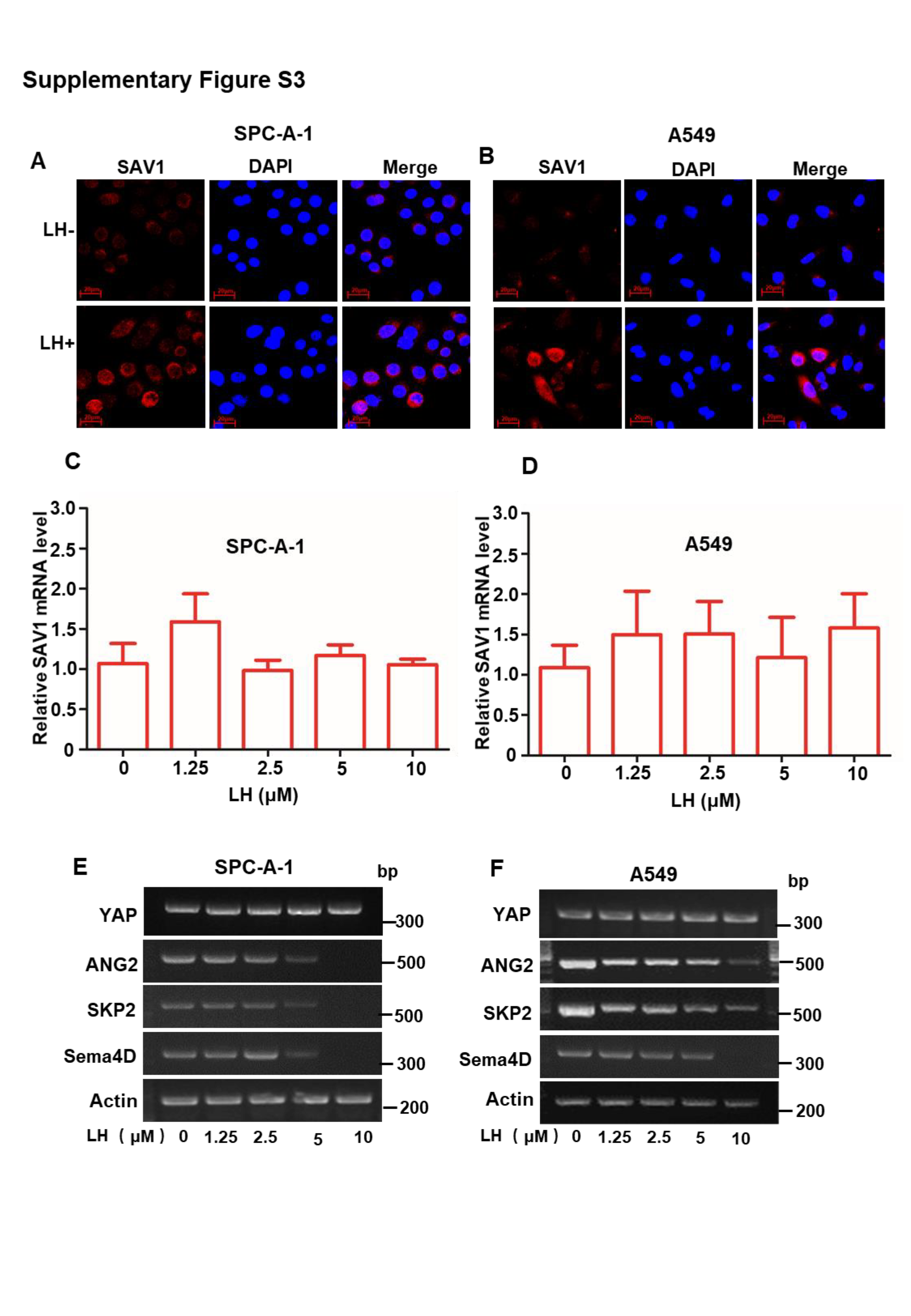

Supplement: Supplementary file 3 — Supplementary figure 3 [file 41419_2020_2591_MOESM3_ESM.tif]

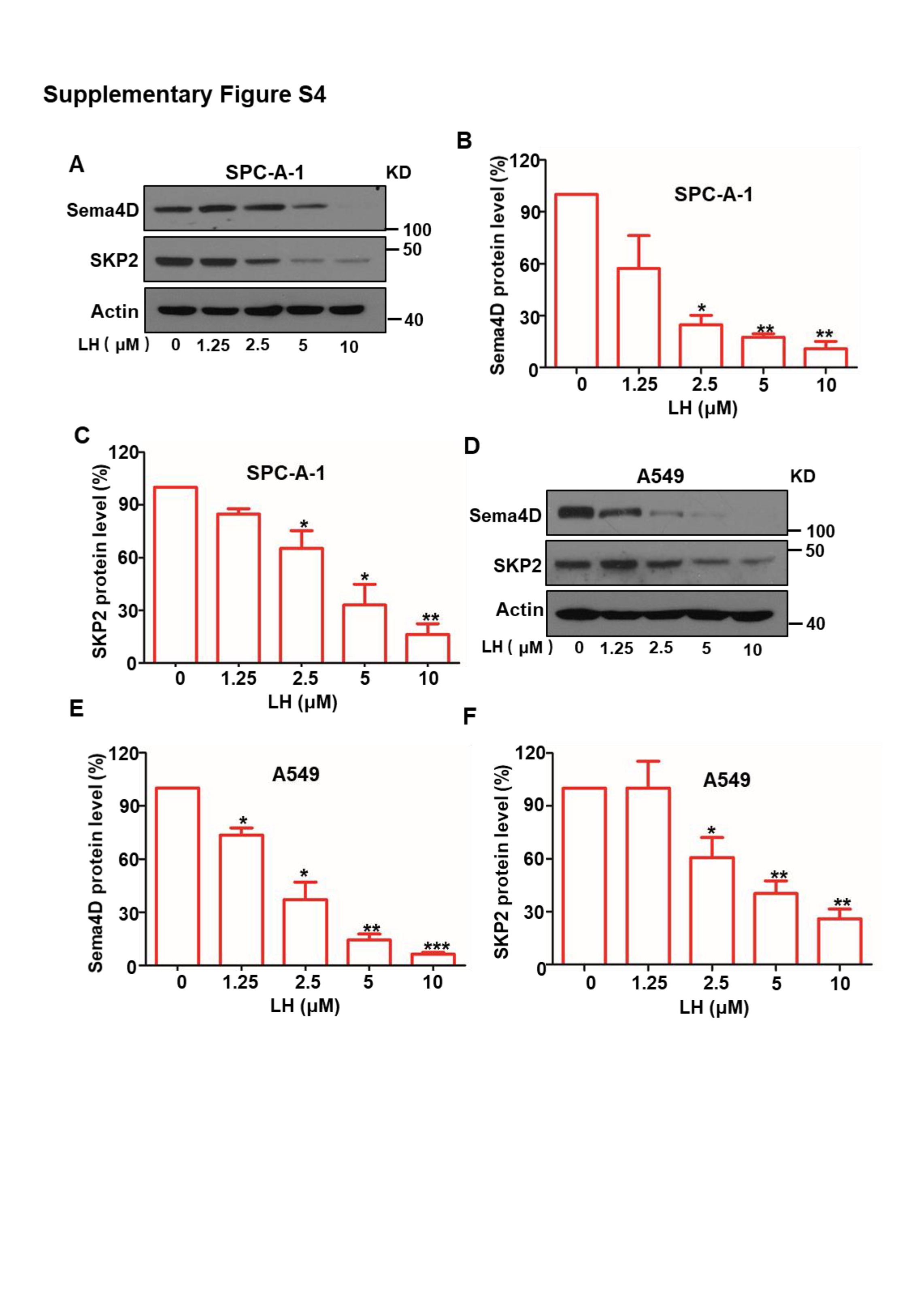

Supplement: Supplementary file 4 — Supplementary figure 4 [file 41419_2020_2591_MOESM4_ESM.tif]
