# Supplementary material for: Correction of the tumor suppressor Salvador homolog-1 deficiency in tumors by lycorine as a new strategy in lung cancer therapy
Source: Cell Death Dis. 2020 May 21;11(5):387. doi: 10.1038/s41419-020-2591-0 (PMC7242319; doi:10.1038/s41419-020-2591-0)
Supplement: Supplementary file 5 — Supplementary figure legends [file 41419_2020_2591_MOESM5_ESM.docx]

**Supplementary figures**

**Supplementary Figure S1. Lycorine inhibited lung cancer cell proliferation and induced cell apoptosis.**

Lycorine inhibited the proliferation of the lung cancer SPC-A-1 and A549 cells, (Supplementary Figure S1A, S1B) in a concentration dependent manner, but was less sensitive to the immortalized normal HBE cells (Supplementary Figure S1C). The cell cycle assay indicated that lycorine arrested cancer cell cycle at G2/M phase (Supplementary Figure S1D, S1E). Lycorine significantly induced lung cancer cell apoptosis at the concentrations of 20 and 40 μM (Supplementary Figure S1F). Data are shown as mean ± SD of three independent replicates.

**Supplementary Figure S2. Lycorine does not significantly affect mouse body weight, organ coefficients, and SAV1 and YAP mRNA levels.**

The mouse body weight and organ coefficients in three types of mouse models were analyzed. Lycorine did not significantly affect the body weight in subcutaneous neoplasia (Supplementary Figure S2A) and lung metastasis (Supplementary Figure S2B), and the *vivo* Matrigel plug assay (Supplementary Figure S2D) mouse models, meanwhile, there were no difference in the mouse organ coefficients between the lycorine-treated and the control mice (Supplementary Figure S2C). Quantitative real time PCR showed that the SAV1 and YAP mRNA levels were not changed in the tumors from the tumor bearing mice after lycorine treatment (Supplementary Figure S2E). Data are shown as mean ± SD of three replicates.

**Supplementary Figure S3. Influence of lycorine on the expression of SAV1 and YAP-activated transcription of oncogenic genes in lung cancer cells.**

Immunofluorescence staining (IF) and confocal microscopy imaging immunostaining showed that lycorine enhanced SAV1 expression in SPC-A-1 (Supplementary Figure S3A) and A549 (Supplementary Figure S3B). However, SAV1 mRNA levels in SPC-A-1 ((Supplementary Figure S3C) and A549 cells (Supplementary Figure S3D) were not significantly changed after lycorine treatment. Additionally, YAP-mediated expression of the oncogenic genes ANG2, SKP2 and sema4D, was significantly reduced by lycorine in a concentration dependent manner in SPC-A-1 (Supplementary Figure S3E) and A549 cells (Supplementary Figure S3F), while YAP protein was marked reduced by lycorine (see main Figure 4A, 4B), YAP mRNA was not influenced with lycorine treatment (Figure S3E, S3F).

**Supplementary Figure S4. Lycorine reduced the levels of Sema4D and SPK2 proteins in lung cancer cells.**

Western blotting showed that lycorine decreased sema4D and SKP2 protein levels in SPC-A-1 (Supplementary Figure S4A-4C) and A549 (Supplementary Figure S4D-4F) in a concentration dependent manner. (Figure S4A-4F).
